# Assessing labor market impacts of trade opening in Uruguay

**DOI:** 10.1186/2193-1801-2-219

**Published:** 2013-05-10

**Authors:** Adriana Peluffo

**Affiliations:** Instituto de Economia, Universidad de la Republica, Dr. Joaquin Requena, 1375 Montevideo, Uruguay

**Keywords:** Trade, Labor markets, Employment, Wages, Trade and labor market interactions

## Abstract

F02, F16, J23, J31

## Introduction

The analysis of the impact of increasing globalization on labor markets has been a focus of research in the last decades. Initially studies focused on developed countries and analyzed how opening to trade would affect workers with different skills (Freeman 1995; Feenstra and Hanson 1999). As developing countries start to open up their economies and data become available these countries also turn out to be a focus of analysis. Nevertheless, so far the result are not clear cut, and there is mixed evidence on the effects of trade liberalization on labor markets. One of the most puzzling findings is that there is evidence of a skill-bias in labor demand and increased wage inequality as a result of increasing trade liberalization, both for developed and developing countries (Attanasio, Goldberg, and Pavcnik 2004; Feenstra and Hanson 1997; Robbins 1996; Perry and Olarreaga 2007).

In this work we propose to analyze the impact of the increase in trade liberalization in Uruguay, as a consequence of Mercosur’s creation on wages, unemployment and wage dispersion at the individual level. To this aim we apply impact evaluation techniques using data from the Encuesta Continua de Hogares (ECH) for the period 1988 and 1996, i.e. before and after Mercosur’s creation.

A contribution of this paper is the use of the difference-in-difference approach, which is not common in trade empirical works, to analyze the impact of trade liberalization at the household level for a small developing country. In particular, the matching and double difference (MDID) approach has the advantage of removing the effects of common shocks. This make possible to isolate the effect of trade reforms from other policies during the period, providing in this manner a more accurate analysis of the impact of trade openness.

This work structures as follows: after the introduction, in section 2 we present some background literature. In section 3 we describe briefly some features of the trade liberalization process in Uruguay. In the fourth section we present the empirical implementation. In the fifth section we present the results and finally some concluding remarks.

## Literature review

It is worth devoting some words to the links between trade policy and labor market outcomes. Trade policies can have a significant impact on the level and structure of employment, on wages and wage differentials, and on labor market institutions and policies. Nevertheless labor and social policies can also influence the outcomes of trade policies in terms of growth of output, employment and the distribution of income.^a^

Trade liberalization is associated with both job destruction and job creation. The net employment effect in the short run depends mainly on country specific factors such as the functioning of the labor market. In the long run, the efficiency gains due to trade liberalization are expected to generate positive employment effects, either in terms of quantity or quality of jobs or a combination of both.

The theoretical literature provides insights into the process of job destruction and job creation following trade liberalization and illustrates how different country characteristics can affect temporary and permanent employment at the sectoral or country level (Lee, Vivarelli and Office 2006).

The classical link between trade and income inequality is based on the Stolper-Samuelson Theorem developed in a model that assumed full employment. According to this theorem inequality is most likely to increase in industrialized countries as a consequence of trade with developing countries because the former are well endowed with skilled labor. While in developing countries is expected to observe a decline in inequality. This would happen because developing countries are typically well endowed with low skill labor relative to developed countries. With a move to free trade, developing countries will be more competitive in low skill intensive sectors which will expand. The increased demand for low skilled workers, who typically belong to the poorer segments of the population, will lead to an increase in their wages relative to the wages of skilled workers. Thus, the theoretical literature predicts that trade liberalization raises average income levels, and also some contributions of the theoretical growth literature suggest that trade also stimulates growth.^b^

Traditional trade models assume full employment, though some workers may be better or worse off in the long run due to changes in wages. It is assumed that on average, individuals would be better off as a result of overall efficiency gains triggered by trade liberalization. However, many economies are not characterized by full employment.^c^ In this case trade liberalization would reduce demand for workers mainly in import competing sectors and unemployment would increase.

Recent trade models point out that adjustment processes may not only be observed between sectors but also within sectors. The “new-new trade models” that introduce firm heterogeneity and fixed-market entry costs predict that trade reform will trigger job creation and job destruction in all sectors, as both net-exporting and net-importing sectors will be characterized by expanding high-productivity firms and low-productivity firms that will shrink or close down. This implies that an important reshuffling of jobs takes place within sectors.

Even though the economic literature has produced a large number of empirical studies analyzing the effects of trade on labor market outcomes, so far no clear message emerges from this literature.

Milner and Wright (1998) for Mauritius find increases in employment after trade liberalization while Harrison and Revenga (1995) for several developing and transitional countries find increases in employment for developing countries and reductions for transitional economies.

Regarding to wages the works by Rama (2003) and Lopez (2004) find short-run reductions in wages after trade liberalization but positive long run effects, i.e. increases in wages in the long run which are attributed to growth effects.

Most empirical works for Latin America suggest that trade liberalization has led to an increase in both income and wage inequality and a skill bias of labor demand (Robbins 1996; Attanasio, Goldberg and Pavcnik 2004; Feenstra and Hanson 1997; Perry and Olarreaga 2007; Barraud 2009; Wood 1997; Slaughter 2000). Dollar and Kraay (2004) find that trade openness affects income distribution positively. A similar result is obtained by Behrman, Birdsall and Székely (2000) for a set of Latin American countries. Dollar and Kraay (2004) find that trade openness affects income distribution positively. A similar result is obtained by Behrman, Birdsall and Székely (2000) for a set of Latin American countries. However, Sanchez-Paramo and Schady (2003) find the opposite result in six Latin American countries, where trade volumes would negatively affect inequality. Spilimbergo et al. (1999) also find that trade openness would be associated with higher inequality, whereas Edwards (1998) does not find any significant effect of trade on income distribution. Galiani and Porto (2006) find a negative effect of tariff reforms on the wage levels in Argentina.^d^ More recently Barraud (2009) analyzing the effect of trade liberalization on wages for Argentina using difference-in-differences and matching techniques, finds that labor market and poverty indicators deteriorated in the 1988–1998 liberalization period in Argentina.

For the Uruguayan case Casacuberta and Vaillant (2002) find that the higher the tariff reduction the higher was the reduction in employment and wages at the industry level in the 90s. Galiani and Sanguinetti (2003) working for the same period, find that Mercosur trade flows have negatively affected the level of industry employment in Uruguay. However these results are obtain trough correlations so they are not controlling for other forces that may have induced different manufacturing activities to change their employment levels.

The whole picture that emerges is that this literature does not appear to allow for any general conclusion on the link between trade liberalization and income distribution and the impression arises that this link is country and situation specific.^e^

Hence, so far empirical research into the link between trade liberalization and market labor outcomes has produced mixed results. While the evidence for Asia seems to confirm a reduction in inequality following trade liberalization in Latin America inequality seems to have increased.

The only general conclusion that may be justified is that employment effects depend on a large number of country-specific factors, aside differences in the quality of the data and econometric issues of the studies.

One shortcoming of the studies is that they fail to distinguish the different possible causes of employment changes. Labor market policies, macroeconomic policies, technological change or movements along the business cycle are only a few examples of factors that may affect an economy’s employment level.^f^

As we have already mentioned we should keep in mind the difficulty of isolating the effects of trade from other policies implemented simultaneously with trade reform. In most studies, the identification of trade effects relies on the comparison before and after a policy change without controlling for other policies at work. As a consequence, this approach attributes changes originating from other sources to trade policy. Most studies use data covering only a short time period after the reform which implies that the results can be heavily affected by the cyclical behavior of the economy. The difference-in-difference methodology should eliminate the effects of common shocks providing so a more precise description of the impact of trade policy as we explain in Section 4. We try to improve over Barraud’s study by using a double-robust estimator which allows obtaining unbiased estimates when there are confounding factors (e.g. changes in technology, in labor supply, in institutional settings and other policies that may affect the outcome as well as the probability of treatment).

## Trade openness in Uruguay

As most countries in the region, Uruguay has pursued an import substitution policy from the early 1930s to mid-70s (Bértola 1991). Since 1974 trade policy has been characterized by a continuous reduction in tariff barriers, both in the number of tariffs levels and in average rates. Also non-tariff barriers were eliminated, remaining mainly References Prices and Minimum Export Prices. Nevertheless, the number of goods subject to Reference Prices and Minimum Export Prices experienced a dramatic reduction.^g^

In 1991 Uruguay signed the Asuncion Treaty aimed to the creation of the Southern Common Market (MERCOSUR) with Argentina, Brazil and Paraguay, which implied a deepening in the liberalization process.^h^ In 1995 the custom union was functioning for the 85 per cent of the tariff lines, though the four countries have kept a list of exemptions for some goods, and it was still an imperfect custom union.

The integration process was verified in a context of the return to democracy and growth in the region, along with policies of trade and financial liberalization and stabilization. The creation of the MERCOSUR decreases the cost of access to partners’ markets and could imply an enlargement of the market, which could allow reaching economies of scale. Nevertheless, the degree of development and economic size between countries and regions is very uneven which may act as an impediment to deeper integration.

After MERCOSUR creation there has been an important rise of Uruguayan trade with the big partners: Argentina and Brazil, for both imported and exported goods. The average values for the period 1975–1978 show that exports to Argentina and Brazil were 22 per cent of total Uruguayan exports while this figure raise to 46 per cent in the period 1994–1996.^i^

In 1995 the tariff structure of the bloc was adopted. Thus, the protection levels in Uruguay, regarding extra-regional trade are defined basically through two key instruments: tariffs and the exchange rate.

On the other hand, the exchange rate was used as an instrument to reduce inflation, and domestic currency was strongly appreciated during most of the period analyzed. In the 90s the policy designed to reduce inflation was to tie the peso to the dollar (crawling peg or “ancla cambiaria”). The Stabilization Plan was triggered by an inflation that reached the three digits, in January 1991, which was implemented in 1992. The monetary aspect of the plan was the use of the domestic currency pegged to the dollar. This was instrumented by the Central Bank through a band regime with pre-announced ex-change rates. This policy was successful in reducing inflation which fell steadily since 1991 up to the year 2002. The cost of this policy was to make exports less competitive, mainly outside the region, since Brazil and Argentina also implemented similar stabilization policies.^j^

In Table 1 we present the evolution of manufacturing gross product, imports and exports for the period 1988 up to 2001, while in Table 2 we present the evolution of the Openness Index and the Import Penetration and Export ratio. We can observe the increase in openness in the three indicators considered. They increase steadily up to 2000 and contract in 2001 along with the Argentinean crisis.

**Table 1:** **Evolution of GDP, imports and exports (thousands of constant pesos base year 1983)**

Year	GDP	Imports	Exports
1988	209,892	52,321	51,373
1989	212,209	54,909	55,228
1990	212,840	54,548	62,795
1991	220,372	64,409	64,504
1992	237,851	80,591	70,387
1993	244,142	94,473	76,459
1994	261,951	111,734	88,038
1995	258,159	108,341	86,403
1996	272,559	120,617	95,287
1997	286,317	136,593	107,695
1998	299,311	147,013	108,055
1999	290,791	138,503	100,099
2000	286,600	138,600	106,467
2001	276,898	128,785	96,748

*GDP*: gross domestic product, *M*: imports, *X*: exports.

Source: Own elaboration based on data from the Uruguayan Central Bank (Banco Central del Uruguay).

**Table 2:** **Evolution of the openness coefficient (OI) and import penetration (IP) and exports ratio**

Year	0I	IP	X/GDP
1988	0.49	0.25	0.24
1989	0.52	0.26	0.26
1990	0.55	0.26	0.30
1991	0.58	0.29	0.29
1992	0.63	0.34	0.30
1993	0.70	0.39	0.31
1994	0.76	0.43	0 34
1995	0.75	0.42	0 33
1996	0.79	0.44	0 35
1997	0.85	0.48	0 38
1998	0.85	0.49	0 36
1999	0.82	0.48	0 34
2000	0.86	0,48	0 37
2001	0.81	0.47	0 35

*OI*: openness coefficient defined as (Exports + Imports)/GDP and IP: import penetration ratio defined as M/GDP and the export ratio is defined as Exports/GDP.

Source: own elaboration based on data from the Uruguayan Central Bank (Banco Central del Uruguay).

Regarding to labor market institutions, during the military coup in the country (1973–1984) collective bargaining was proscribed, but labor unions regained the right to bargain collectively with the return to democracy in 1985. As part of its anti-inflationary policy, the national government –Sanguinetti’s administration- played a significant role in negotiations. Since then, labor regulations can be superseded by collective agreements. They can go beyond these restrictions increasing the benefits that workers have in the area of minimum wages, working conditions, job security and employment benefits. Tripartite negotiations took place at the industry level through “Wage Councils”, allowing wage adjustment to vary by industry. If an agreement met the government anti-inflationary target, then it would apply to all firms in the industry –even those with non-union workforces- once the agreement was officially endorsed.

In 1991 –Lacalle’s administration- the government stopped participating in this system, though some bargaining was still conducted through industry wide Wage Councils, but was increasingly done at the firm level. By 1992 the contracts signed under the Wage Council system have expired. Furthermore, union density fell from 42 percent to 22 percent in 1993 and stayed in that level since then (Cassoni et al. 2004).

In summary, under the period analyzed in this work there was two different bargaining regimes: 1)1985-1991 when there was a tripartite bargaining; and 2) from 1992 till 2005, when the government did not participate in bargaining.

By the end of the 90s the share of manufacturing product in gross domestic product (GDP) as well as the number of manufacturing firms has decreased substantially and the unemployment rose. In Table 3 we present the share of manufacturing product in GDP, while in Table 4 we present the employment rate, the number of persons employed and the unemployment rate and the number of persons unemployed. We can observe an increase in unemployment in the Uruguayan economy during the period.

**Table 3:** **Share of manufacturing product in GDP**

Year	GDP	MGP*	MGP/GDP
1988	209,892	55,667	0.27
1989	212,209	55,560	0.26
1990	212,840	54,750	0.26
1991	220,372	54,464	0.25
1992	237,851	50,328	0.21
1993	244,142	55,296	0.23
1994	261,951	50,361	0.19
1995	258,159	50,877	0.20
1996	272,559	52,918	0.19
1997	286,317	56,023	0.20
1998	299,311	57,330	0.19
1999	290,791	52,514	0.18
2000	286,600	51,424	0.18
2001	276,898	47,537	0.17

*MGP: Manufacturing gross product in thousands of constant pesos, base year 1983.

Source: Own elaboration based on data from the Uruguayan Central Bank (Banco Central del Uruguay).

**Table 4:** **Employment and unemployment in Uruguay, 1988-2001**

Year	Employment rate (%)	No. of persons employed (thousands)	Unemployment rate (%)	No. of persons unemployed (thousands)
1988	52.2	1,077.30	8.6	101.9
1989	53.1	1,108.40	8	95.2
1990	53.5	1,110.60	8.5	102.1
1991	523	1,125.40	8.9	109.9
1992	52.2	1,14290	9	113.2
1993	52	1,156.00	8.3	105.4
1994	52.8	1,18690	9.2	121.1
1995	53	1,206.00	10.3	137.5
1996	513	1,174.80	11.9	159.1
1997	51	1,172.40	11.4	151.5
1998	54.3	1,10310	10.1	123.8
1999	52.6	1,082.10	11.3	137.7
2000	51.5	1,067.60	13.6	167.7
2001	51.4	1,076.20	15.3	193.2

Source: Own elaboration based on data from the Instituto Nacional de Estadísticas (INE).

## Empirical implementation

### Methodology

This paper use a difference-in-differences methodology which allows to study the impact of increased trade exposure due to the creation of the Mercosur (the treatment) on the tradable sector (treated group) relative to individuals working in industries that did not increase their exposure to foreign competition (control group). We estimate regressions equations in double differences without matching as well as matching and double-differences.

#### Regression equations (DID)

In the case of regression equations our baseline equation to estimate is the following:

1 $$Y_{\mathrm{it}}=\beta_{o}+\beta_{L}TT_{\mathrm{it}}+\beta_{x}X_{\mathrm{it}}+D_{j}+d_{t}+\epsilon_{\mathrm{it}}$$

where *Y*_*it*_ is the outcome for household *i* at time *t*. As outcome variables we consider monthly labor earnings, total hourly labor earnings, hourly wages, hourly labor earnings in monetary terms and in-kind, unemployment probability and wage dispersion. *TT*_*it*_ is the trade liberalization variable on the treated individuals, i.e. the treatment on the treated. It is constructed by interacting individuals working in the manufacturing industries (*Lib*_*it*_, where manufacturing = 1 and service or control group = 0) with a time dummy that takes the value of one for 1996 (five years after the creation of the MERCOSUR). Dj stands for industry dummies and dt for a time dummy.

As treatment group we consider those individuals working in the manufacturing (tradable) sector while as non-tradable we consider the public employees as explained below.

*X*_*it*_ is a set of control variables or covariates which includes age, marital status, head of the household, sex, hours worked the week before to the survey, and schooling years.

As we mention before, to construct the liberalization variable (*TT*_*it*_), we define the treated group as those individuals working in the tradable industries (*Lib*_*it*_) after MERCOSUR’s creation. Our control group is integrated by individuals working in the public sector, which are likely to be less affected by trade openness. We should note that this definition of the tradable and non-tradable groups is not free of criticism, namely since it could exist contagion between both groups. In this regard, Barraud and Calfat (2008) analyzing the effect of trade liberalization on wages for Argentina find evidence of significant impacts of trade liberalization on several non-tradable sectors as well as an important shift of manufacturing workers to services, which would indicate that the service sector is also likely to be affected by trade liberalization.

Nevertheless, using public employees as control group has the advantage that they have different characteristics relative to the private sector employees regarding to the conditions of entry, the possibilities of lay-offs, and the setting of their wages. Further, from the data we observed that unemployment rates for the control group selected is significantly lower than for the manufacturing one. In this regard, we analyze the proportion of unemployment by group finding that 74% comes from manufacturing and 26% from the service sector, corroborating the assumption that manufacturing is by large more affected due to trade liberalization than the control group.^k^

Another reason for choosing public servants as control group, related to the above, is that there is a lower inter-sectoral mobility between both groups since there are institutional and traditional barriers to entry in the public sector, preventing workers from the industrial sector to move freely into some government jobs. Furthermore labor skills are usually specific to each sector, and this would also be a factor restricting mobility between both groups. Aside these characteristics, which could help to prevent contagion, we can assume that public employees share a similar set of characteristics with those working in the manufacturing sector, that is to say, the individuals in the treatment group.^l^

Finally, in a similar work for the Argentinean case Barraud (2009) uses services as control group.

We also tried different definitions of control groups based on different aggregations of public workers according to the sector in which they work as shown in Table 5.

**Table 5:** **Definitions of control groups**

Variable	Definition
emp_pub1	Public Workers in all sectors
emp_pub2	Public workers in the division “Community Social and Personal Services” – excludes public enterprises
emp_pub3	Public workers in the division “Community Social and Personal Services – excludes public enterprises – and “Personal and Household Services” as well as “International Organizations”
emp_pub4	Public workers “Public Administration and Defense“ and ”Social and other community and related services”
emp_pub5	Public workers in “Social and other community and related services”

Source: Own elaboration.

Thus, we work with civil servants in Public Administration and Defense and “Social and other community and related services” (emp_pub4) as control group, since it has enough number of observations to conduct the analysis and excludes public workers that belong to industries that were affected by increased trade openness such as some public enterprises as refineries (ANCAP) and financial services.

Variables were deflated by the price deflator so they are expressed in real terms with base in December 2006.

#### Matching and double differences

The effect of Mercosur’s creation is the estimated difference-in-difference of the outcome variable (earnings, hourly wage, probability of unemployment and wage dispersion) between the treated and the control groups. The difference-in-difference methodology is implemented using a double-robust estimator with inverse probability weighting and difference-in-differences estimation. We use a matching and difference-in-differences methodology which allows studying the causal effect of increasing trade liberalization (the treatment) on individuals which belong to the tradable group (the treated) relative to individuals that were not –or at least into a lower extent- affected by the opening of the economy (the control group). Thus, our aim is to evaluate the causal effect the creation of the Mercosur on **Y**, where **Y** represent monthly earnings, total hourly labor earnings, hourly wages, unemployment probability and wage dispersion. Y is referred to as the “outcome” in the evaluation literature.^m^

Let increased trade openness due to Mercosur’s creation (TT) where TT_it_ ∈ {0, 1} denotes an indicator (dummy variable) of whether household *i* has been exposed to increased trade openness- and $Y_{i,t+s}^{1}$ is the outcome at *t + s*, after Mercosur’s creation, i.e. after 1991. Also denote by $Y_{i,t+s}^{0}$ the outcome of household *i* had it not been exposed to increased trade openness. The causal effect of the treatment on the treated (TT) for household *i* at period (t + s), and the response variable Y, be defined as:

$$Y_{i,t+s}^{1}-Y_{i,t+s}^{0}$$

The fundamental problem of causal inference is that the quantity $Y_{i,t+s}^{0}$, referred as the counterfactual, is unobservable. Causal inference relies on the construction of the counterfactual, which is the outcome the individuals would have experienced on average had they not been exposed to increased trade openness. The counterfactual is estimated by the corresponding average value of individuals that do not have experienced an increased in trade exposure. An important issue in the construction of the counterfactual is the selection of a valid control group and to this end me make use of matching techniques.

The basic idea of matching is to select from the group of individuals belonging to the control group those in which the distribution of the variables **X**_it_ affecting the outcome is as similar as possible to the distribution of the individuals belonging to the treated group. The matching procedure consists on linking each treated individual with the same values of the **X**_it_. We adopt two different matching methods which make use of the “propensity score matching”. To this end, we first identify the probability of being exposed to increased trade openness (the “propensity score”) for all individuals, irrespective if they belong to the treated or control group by means of a logit model. There are several matching techniques, and in this work we use double-robust estimators with inverse probability weighting. Inverse probability weighting derives weights from the propensity score, where these weights are defined by the inverse of the propensity score if the individual receives treatment and the inverse of one minus the propensity score if the individual is in the control group. Further, inverse probability weighting should mitigate the problem when there are not common trends between the treated and the control group.

A matching procedure is preferable to randomly or arbitrarily choosing the comparison group because it is less likely to suffer from selection bias by picking up individuals with markedly different characteristics.

As Blundell and Costa Dias (2002) point out, a combination of matching and difference-in-difference is likely to improve the quality of non-experimental evaluation studies. The difference-in-difference approach is a two-step procedure. Firstly, the difference between the average outcome variable before and after the treatment is estimated for firms belonging to the treated group, conditional on a set of covariates **(X**_**it**_**)**. However, this difference cannot be attributed only to increased trade openness since the outcome variables might be affected by other macroeconomic factors, such as policies aimed to stabilization of the economy. To deal with this the difference obtained at the first stage is further differenced with respect to the before and after difference for the control group.

The difference-in-difference estimator therefore removes effects of common shocks and provides a more accurate description of the impact of the trade liberalization on labor markets. Furthermore, the double-robust estimator offers increased protection against model misspecification and also gives unbiased estimates of the treatment effect when the models are correctly specified, allowing so to obtain more accurate results (Emsley et al. 2008).

### Data sources and variables

Data come from the Continuous Household Survey (ECH) for the years 1988 and 1996, recorded by the Instituto Nacional de Estadística (INE). These surveys are representative of urban areas with more than 900 inhabitants in the whole country.

Due to data availability we work with the main occupation. Also, for the sake of simplicity we consider only the employees and drop from the sample owners, independents workers and cooperative members, and individuals with less than 18 years and more than 65 years. Thus, the treated group is defined as those individuals whose main occupation is as workers in Manufactures while the control group is composed by public workers in “Public Administration and Defense” and “Social and other community and related services” as main occupation.^n^ In Table 6 we present the number of observation in each group and year.

**Table 6:** **Number and percentage of observations in the control and treated groups– 1988 and 1996, urban areas with more than 900 inhabitants**

	***1988***		***1996***	
	Observations	%	Observations	%
Control group	3863	51.3	3054	53.7
Treated group	3667	48.7	2631	46.3
Total	7530	100	5685	100

Source: Own elaboration based on ECH 1988 and 1996.

As can be observed from Table 6, the control group represents a slightly higher number of observations of the total sample for the two years considered in this work. The outcome variables considered regarding earnings and wages are monthly earnings, total hourly labor earnings –including leave pays and bonuses-, hourly wages and hourly earnings (monetary and in-inkind and excluding leave pays and bonuses). As we have mentioned before all the income variables are in real terms with base December 2006. Furthermore we analyze the probability of unemployment, estimated through a logit model,° and wage dispersion.

Monthly labor earnings are composed by all the net incomes from the main occupation, i.e. monetary wages and wages in-kind, commissions, bonuses, leave pays, tips and others compensations. To estimate the total hourly labor earnings we divide monthly earnings by the hours worked in the week in the main occupation, i.e. the one that represents the main earnings.^p^ Hourly wages considers exclusively the monetary earnings (wage and commissions) per hour in the main occupation in monetary terms, while hourly labor earnings takes into account wages and earnings in-kind and exclude bonuses and holiday pays.

In order to analyze wage inequality we defined the average value of the 80th percentile of total hourly wages and monthly labor earnings^q^ and compute the ratio of these values over hourly labor earnings and monthly labor earnings for each individual generating the variables named gap2 and gap3 respectively at the 2 digit ISIC level.^r^

In Table 7 we summarize the main features of the variables considered in this work for the years 1988 and 1996 while in Table 8 we present total hourly and income wage gaps.

**Table 7:** **Descriptive statistics– 1988 and 1996, urban areas with more than 900 inhabitants**

		1988								
		Total			Treated			Non-treated		
		No. Obs.	Average	S.E.	No. Obs.	Average	S.E.	No. Obs.	Average	S.E.
Outcome Variables	Monthly earnings	7530	10741.35	9024.929	3667	11043.48	10811.73	3863	10454.55	6903.341
	Total hourly labor earnings*	7530	60.9388	58.64012	3667	56.18011	65.61545	3863	65.45604	50.74353
	Hourly wages (hourly monetary wage)	7530	51.93759	49.93657	3667	48.62609	57.96903	3863	55.08107	40.63559
	Hourly labor earnings (monetary and in-kind)**	7530	54.37341	50.49756	3667	49.80618	58.25184	3863	58.70891	41.37124
	Unemployment probability	7530	0.0692962	0.0619908	3667	0.0811074	0.073021	3863	0.0580843	0.0466049
Control Variables	Montevideo	7530	0.4742364	0.499369	3667	0.5396782	0.4984911	3863	0.4121149	0.4922793
	Sex (man = 1)	7530	0.6258964	0.4839227	3667	0.6817562	0.4658582	3863	0.5728708	0.4947254
	Age	7530	37.39867	11.66889	3667	36.18871	12.28136	3863	38.54724	10.93454
	Squared Age	7530	1534.806	928.4611	3667	1460.413	959.5702	3863	1605.423	892.3375
	Head of household	7530	0.5017264	0.5000302	3667	0.4935915	0.5000271	3863	0.5094486	0.4999754
	Schooling years	7530	9.2	4.156597	3667	7.862285	3.148509	3863	10.46984	4.57823
	Hours worked	7530	44.49588	15.3459	3667	47.36215	12.90632	3863	41.77505	16.90496
	Number of jobs	7530	1.133599	0.3818153	3667	1.065176	0.2566215	3863	1.19855	0.4615489
		**1996**								
		**Total**			**Treated**			**Non-Treated**		
		**No. Obs.**	**Average**	**S.E.**	**No. Obs.**	**Average**	**S.E.**	**No. Obs.**	**Average**	**S.E.**
Outcome Variables	Monthly earnings	5685	11577.71	10108.74	2631	12139.73	12448.73	3054	11093.54	7499.512
	Total hourly labor earnings	5685	68.16526	59.86078	2631	62.94509	63.4181	3054	72.66239	56.24047
	Hourly monetary wages (hourly monetary wage)	5685	57.16029	52.29815	2631	53.01425	54.94132	3054	60.73208	49.64109
	Hourly labor earnings (monetary and in-kind)	5685	62.68532	53.21773	2631	57.97227	55.72992	3054	66.74558	50.61269
	Unemployment probability	5666	0.0938942	0.072972	2625	0.1156789	0.0840809	3041	0.0750897	0.0552922
Control Variables	Montevideo	5685	0.517854	0.4997251	2631	0.5549221	0.4970689	3054	0.4859201	0.4998836
	Sex (man = 1)	5685	0.5825858	0.4931758	2631	0.6913721	0.462015	3054	0.4888671	0.4999579
	Age	5685	38.24468	11.72058	2631	35.75067	12.20706	3054	40.39325	10.83565
	Squared Age	5685	1600.003	932.6359	2631	1427.066	945.2063	3054	1748.988	895.4232
	Head of household	5685	0.4666667	0.4989315	2631	0.4583808	0.4983596	3054	0.4738048	0.4993951
	Schooling years	5666	9.965055	3.963805	2625	8.590476	3.123522	3041	11.15159	4.221075
	Hours worked	5685	42.26684	14.08965	2631	45.75675	10.95456	3054	39.26031	15.70613
	Number of jobs	5683	1.146402	0.3967028	2631	1.066515	0.2625988	3052	1.215269	0.4726435

*Include leave pays and bonuses; **Excludes leave pays and bonuses; S.E.: standard errors.

Source: Own elaboration based on ECH 1988 and 1996.

**Table 8:** **Hourly and total income wage gaps**

Hourly wage gap (gap2)	1988	1996
Average	2.965	3.781
Standard Dev.	2.668	29.71
No. Observations	7530	5681
**Monthly labor earnings gap (gap3)**		
Average	2.88	3.625
Standard Dev.	3.722	22.46
No. Observations	7530	5681

Source: Own elaboration based on ECH 1988 and 1996.

We can observe an increase in income and wage gaps in the period with and important decreased in the standard deviation, pointing out to an increased dispersion of these variables.

## Results

### Difference-in-difference regressions

Following the methodology presented en Section 4 we estimate regression equations in double differences in order to estimate the impact of the increased trade openness due to Mercosur’s creation, on a selected group of impact variables regarding labor earnings and wages, unemployment probability and wage dispersion. We choose to focus our analysis mainly for the period 1988–1996, this is five years after the creation of the Southern Common Market, since generally it is assumed that the initial shock and the reallocation of resources due to trade liberalization will operate in a four/five year period, while dynamic effects will take a longer period to be felt.

Firstly we analyze the results obtained for earnings and wages: monthly earnings, total hourly labor earnings, hourly wages, and hourly monetary and in-kind earnings. From the *t*-test analysis^s^ we can observe that monthly earnings are significantly higher for the treated than for the control group in both years, while hourly wages are significantly higher in both years for the control group and the unemployment rate is significantly higher in the treated for both years. In Table 9 we present the results for the difference-in-differences regressions without matching.

**Table 9:** **Results of the estimation of regression equations in double differences, 1988-1996**

Variables	Monthly earnings	Total hourly earnings	Hourly monetary wage	Hourly labor earnings	Unemployment probability	Ln gap2	Ln gap3
Treatment	0.0624***	0.0137	0.0292*	0.0282*	0.0124***	0.0760***	0.156***
	(0.0161)	(0.0165)	(0.0167)	(0.0160)	(0.0007)	(0.0131)	(0.0134)
Montevideo	0.192***	0.184***	0.167***	0.176***	0.0127***	−0.180***	−0.171***
	(0.0093)	(0.0096)	(0.0096)	(0.0093)	(0.0004)	(0.00923)	(0.00940)
Sex	0.217***	0.110***	0.102***	0.122***	−0.0358***	−0.201***	−0.161***
	(0.0126)	(0.0126)	(0.0121)	(0.0117)	(0.0007)	(0.0124)	(0.0127)
Age	0.0467***	0.0426***	0.0378***	0.0386***	−0.0193***	−0.0431***	−0.0510***
	(0.0029)	(0.0029)	(0.0029)	(0.0028)	(0.0002)	(0.00282)	(0.00291)
Age^2	−0.000448***	−0.000379***	−0.000286***	−0.000325***	0.000201***	0.000417***	0.000488***
	(3.60e-05)	(3.67e-05)	(3.59e-05)	(3.49e-05)	(2.05e-06)	(3.54e-05)	(3.65e-05)
Head of household	0.177***	0.139***	0.0787***	0.101***	−0.0321***	−0.182***	−0.166***
	(0.0123)	(0.0126)	(0.0123)	(0.0119)	(0.0006)	(0.0122)	(0.0124)
Schooling years	0.0496***	0.0633***	0.0731***	0.0657***	−0.00382***	−0.0514***	−0.0595***
	(0.0013)	(0.0013)	(0.0013)	(0.0013)	(6.71e-05)	(0.00132)	(0.00134)
Marital status	0.117***	0.117***	0.0820***	0.101***	−0.00132***	0.0119***	−0.00815***
	(0.0106)	(0.0109)	(0.0107)	(0.0104)	(0.0005)	(0.000353)	(0.000377)
Hours worked	0.0101***				3.53e-06	−0.130***	−0.122***
	(0.0004)				(1.56e-05)	(0.0105)	(0.0108)
Industry dummies	Yes	Yes	Yes	Yes	Yes	Yes	Yes
Time dummy	Yes	Yes	Yes	Yes	Yes	Yes	Yes
Constant	6.768***	2.043***	1.880***	2.007***	0.551***	2.268***	3.257***
	(0.0576)	(0.0553)	(0.0537)	(0.0523)	(0.0039)	(0.0565)	(0.0585)
Observations	13,196	13,196	13,144	13,164	13,196	13,192	13,192
R-squared	0.356	0.336	0.346	0.342	0.888	0.364	0.357

*** p < 0.01, ** p < 0.05, * p < 0.1; Lngap2: average of the upper 80th hourly wage over the wage of the individual; lngap3: average of the upper 80th total income over total income of the individual. Robust standard errors in parentheses. Source: Own elaboration based on ECH 1988 and 1996.

Mercosur’s creation shows a positive effect on the monthly earnings, hourly monetary wage and hourly labor earnings of the workers affected by increased trade openness, i.e. those workers in the Manufacturing industry. Nevertheless, the treatment is not significant for total hourly earnings.

The control variables behave as expected and are significant in all the cases. In line with previous studies incomes are higher in Montevideo in relation with the rest of the urban areas, higher for men and head of the household. Furthermore, earnings increase with the schooling years and age. With regard to age it could be observed that the coefficient associated to squared age is negative, which would imply that the effect of age is not linear but quadratic, i.e. as age increases income increases at a decreasing rate as expected.

Regarding to the probability of unemployment the impact of Mercosur’s creation is positive and significant, which implies that increasing trade openness increases unemployment probability. Once again the control variables have the expected signs and are significant except for the variable hours worked, indicating that the probability of unemployment is not associated with the amount of hours worked previously to dismissal. Furthermore, from the estimation of the probability of unemployment we find that it is higher in Montevideo –which concentrates half of the population- and it is lower for males, head of families, married and more educated individuals.^t^

There are various possible reasons behind these results. On one side from the analysis of unemployment probability we observe that individuals with higher education were most likely to keep their jobs, translating so in higher wages for individuals that stayed employed. Furthermore, in the country during the 90s there was a rise in firm’s productivity for those firms that survive increasing trade openness (Casacuberta et al. 2004) which could also explain higher salaries. In this regard Casacuberta et al. (2004) find that the manufacturing sector, in response to reductions in trade barriers, undertook a technological change in favor of more capital intensive technologies. This brought a progressive and systematic increase in average labor productivity, though not in capital productivity. This resulted in increased in total factor productivity during the nineties at an annual average rate higher than 3 percent. Thus, higher competition through tariff reductions, as well as the availability of cheaper and better intermediate and capital goods may be behind the higher productivity. These authors also find significant job destruction rates, which are explained mainly by exiting and downsizing of firms.

Similar results regarding higher unemployment following trade liberalization were found for Argentina (Barraud, 2009) and Brazil (Menezes-Filho et al. 2011). For the Brazilian case increased unemployment is consistent with faster productivity growth than sales expansion, so that output shifts to more productive firms while labor does not –it remains unallocated or shifts to less productive firms-. Galiani and Sanguinetti (2003) find that the levels of employment fell during the nineties for Argentina and Uruguay, and an increase in the skill premium. Further, they find that employment has moved against manufacturing and in favor of services. For Uruguay there was also some growth in employment in some services sectors, in particular those related to financial and services to enterprises, construction, and in a lesser extent electricity, gas and water, and telecommunications –which are not considered in our control group-. Also, in both countries there was a rise in the educational level of the labor force.

On the other hand, the results for wage dispersion point out an increase in dispersion, both for monthly earnings and hourly earnings after Mercosur’s creation. This result can be a consequence of the increase in the skill premium due to the technological modernization in this period. In this regard, it is widely accepted that skilled labor is a complement to more intensive capital technologies, which could explain the rise in the demand for qualified work and the increase in dispersion, issue that we analyze below.

Finally, we perform a placebo test using the years 1986–1987, finding also increases in wages, though with a lower magnitude for monthly earnings, unemployment probability and total income dispersion, and a not significant effects for hourly wage dispersion (Table 10). This would give some confidence that for these variables results would be reliable, though when applying double robust estimation and inverse probability weighting common trends between treated and control groups should not be an issue.

**Table 10:** **Results of the estimation of regression equations in double differences, 1986-1987**

VARIABLES	Monthly earnings	Total hourly earnings	Hourly monetary wage	Hourly labor earnings	Unemployment probability	Lngap2	Lngap3
Treatment	0.0479***	0.0394***	0.112***	0.0594***	0.00319***	−0.00981	0.134***
	(0.0127)	(0.0125)	(0.0122)	(0.0121)	(0.000548)	(0.0125)	(0.0127)
Montevideo	0.159***	0.155***	0.172***	0.179***	0.00589***	−0.157***	−0.148***
	(0.00811)	(0.00800)	(0.00777)	(0.00772)	(0.000342)	(0.00803)	(0.00817)
Sex	0.290***	0.260***	0.229***	0.248***	−0.0452***	−0.273***	−0.241***
	(0.0111)	(0.0109)	(0.0104)	(0.0104)	(0.000604)	(0.0110)	(0.0111)
Age	0.0515***	0.0490***	0.0390***	0.0408***	−0.0192***	−0.0491***	−0.0564***
	(0.00258)	(0.00254)	(0.00245)	(0.00245)	(0.000156)	(0.00254)	(0.00260)
Age^2	−0.000536***	−0.000499***	−0.000352***	−0.000396***	0.000200***	0.000503***	0.000581***
	(3.25e-05)	(3.19e-05)	(3.10e-05)	(3.09e-05)	(1.81e-06)	(3.19e-05)	(3.28e-05)
Head of household	0.133***	0.128***	0.0794***	0.109***	−0.0262***	−0.133***	−0.127***
	(0.0111)	(0.0108)	(0.0102)	(0.0102)	(0.000479)	(0.0109)	(0.0111)
Schooling years	0.0451***	0.0510***	0.0563***	0.0517***	−0.00240***	−0.0499***	−0.0547***
	(0.00116)	(0.00114)	(0.00108)	(0.00109)	(4.89e-05)	(0.00115)	(0.00115)
Marital status	0.00857***	−0.0122***	−0.0145***	−0.0133***	2.05e-05*	0.0119***	−0.00725***
	(0.000316)	(0.000304)	(0.000297)	(0.000291)	(1.22e-05)	(0.000304)	(0.000310)
Hours worked	0.131***	0.138***	0.1000***	0.112***	−0.00300***	−0.138***	−0.136***
	(0.00969)	(0.00950)	(0.00906)	(0.00901)	(0.000405)	(0.00954)	(0.00985)
Industry dummies	−0.104***	−0.133***	−0.0638***	−0.119***	0.00248***	0.0376**	0.160***
	(0.0150)	(0.0148)	(0.0145)	(0.0143)	(0.000612)	(0.0148)	(0.0153)
Time dummy	−0.199***	−0.217***	−0.128***	−0.199***	−0.000682	−0.0713***	0.0305
	(0.0228)	(0.0226)	(0.0216)	(0.0214)	(0.000859)	(0.0226)	(0.0229)
Constant	6.759***	2.506***	2.584***	2.605***	0.545***	2.215***	3.147***
	(0.0511)	(0.0508)	(0.0495)	(0.0490)	(0.00349)	(0.0510)	(0.0515)
Observations	15,296	15,296	15,194	15,222	15,296	15,296	15,296
R-squared	0.347	0.409	0.445	0.430	0.905	0.399	0.378

*** p < 0.01, ** p < 0.05, * p < 0.1; Lngap2: average of the upper 80th hourly wage over the wage of the individual; lngap3: average of the upper 80th total income over total income of the individual. Robust standard errors in parentheses. Source: Own elaboration based on ECH 1988 and 1996.

### Matching and difference-in-difference

We use double robust estimators and inverse probability weighting using the distribution of the treated after the treatment to select the match from the control group. A double-robust estimator allows obtaining unbiased inference when adjusting for selection effects such as confounding^u^ by allowing for different forms of model misspecification. Furthermore, it can also offer increased efficiency when the model is correctly specified (Emsley et al., 2008). We further tested that the balancing tests were satisfied.^v^

As a placebo we tried as control group all public workers (emp_pub1)^w^ which includes workers in the financial sector, services to enterprises, and public enterprises (electricity, gas and water, refineries, and telecommunications state enterprises). Thus, we compare our treated group with a control group that could have some contagion problem since now all public workers are included, not only those working in public administration and defense and social related services –which is our preferred control group-. We expect that, using this control group –which may be most likely affected by contagion- there is still an effect of MERCOSUR, lower than when they compare with our preferred control.We present the results in Table 11, and find that for monthly earnings, hourly labor earning (monetary and in-kind) and hourly wages the effect of treatment is not significant, except for hourly labor earning which shows a lower effect than when using our preferred control group (emp_pub4). On the other hand we find that unemployment probability is relatively similar while wage dispersion variables may be questioned since they are slightly higher.

**Table 11:** **Results of the estimation of matching and difference-in-differences with double-robust estimates and inverse probability weighting, 1988-1996**

Outcome variable	Coefficient	Standard error	z
Monthly earnings	0.0663	0.0137	4.84***
Hourly labor earnings (monetary)	0.0493	0.0136	3.63***
Hourly wages	0.0512	0.0133	3.84***
Hourly labor earnings	0.0512	0.0132	3.87***
Unemployment probability	0.0206	0.0005	39.07***
Dispersion in hourly wages (lngap2)	0.0710	0.0137	5.2***
Dispersion in total income (lngap3)	0.1771	0.0138	12.82***

All variables are in natural logarithms.

*** p < 0.01, ** p < 0.05, * p < 0.1.

Source: Own elaboration base on ECH 1988 and 1996.

Thus using double robust estimators and inverse probability weighting we find a positive effect of Mercosur’s creation on earnings and wages for all the definitions tried, a rise in the probability of unemployment and increases in wage dispersion. The results regarding to the increase in unemployment and increased wage dispersion in manufacturing during the 1990s is consistent with previous empirical works for the country (Vaillant and Casacuberta, 2002; Galiani and Sanguinetti, 2003). Furthermore, in the 90s there was an increase in the skill premium of qualified workers as well as technological modernization that took place during this decade. On the other hand the increase in labor earnings of individuals who have stayed employed may also be consistent with the fact that more educated individuals were in better position to keep their jobs as we show below.

We further analyze the outcomes variables by gender and schooling years. Splitting the sample by gender we find significant increases in earnings for men while for women the effects are not significant or even negative (see Table 12). Thus, it seems that increases in real wages are mainly driven by increases in men wages. Further, unemployment probability and wage dispersion are lower for men compared to women. We also note that women are more educated than men. Taking the average for both years (1988 and 1996) for treated and control groups we find that women in average have 10.89 years of schooling while men only 8.65, with a total average of 9.53 years. In 1988 the average year of schooling for women was 10.56 years –for treated and control groups- while for mean the average was 8.39, and the total average of 9.2 years of schooling. In 1996 there is a moderate rise in years of schooling with an average for women of 11.33 and for men of 8.89 and a total average of 9.82 years of schooling. Further we note that the control group is composed by more educated people (10.46 years of schooling for the control group and 7.86 for the treated in 1988, while in 1996 these figures are of 10.57 and 8.59 respectively).

**Table 12:** **Outcomes according to gender, 1988-1996**

Outcome variables	Females, 88-96			Males, 88-96		
	Coefficient	Standard error	z	Coefficient	Standard error	z
Monthly earnings	−0.0632	0.0391	−1.62	0.1008	0.0150	6.73***
Hourly labor earnings (monetary)	−0.0591	0.0389	−1.52	0.0843	0.0148	5.69***
Hourly wages	−0.1468	0.0378	−3.88***	0.1270	0.0140	9.09***
Hourly labor earnings	−0.0378	0.0371	−1.02	0.1264	0.0137	9.23***
Unemployment probability	0.0121	0.0012	9.91***	0.0198	0.0006	34.52***
Dispersion in hourly wages (lngap2)	0.1384	0.0390	3.55***	0.0198	0.0149	1.32
Dispersion in total income (lngap3)	0.3686	0.0393	9.39***	0.1017	0.0150	6.79***

*** p < 0.01, ** p < 0.05, * p < 0.1.

Source: Own elaboration base on ECH 1988 and 1996.

For schooling years we split the sample for individuals with levels of education higher than the sample median for both years (9 years) and those with lower education. Results are reported in Tables 13 and 14. We find high and significant increases in real wages for more educated individuals, and a lower probability of unemployment that for less educated ones. Moreover, hourly wage dispersion changes sign and becomes negative, pointing out to a reduction in wage dispersion among high skilled workers, while total income dispersions remains positive but lower than for less educated workers. This result is also in line with the technological modernization, the improvements in productivity experienced by surviving firms, which in turn may translate into an increased demand for skilled workers as commented above.

**Table 13:** **Outcomes according to schooling years, 1988-1996**

Outcome variable	More than 9 years of schooling			Less than 9 years of schooling		
	Coefficient	Standard error	z	Coefficient	Standard eError	z
Monthly earnings	0.1791	0.0313	5.73***	0.0144	0.0157	0.92
Hourly labor earnings (monetary)	0.1518	0.0316	4.8***	−0.0009	0.0153	−0.06
Hourly wages	0.1629	0.0307	5.31***	0.0108	0.0154	0.7
Hourly labor earnings	0.1960	0.0301	6.5***	0.0365	0.0148	2.47**
Unemployment probability	0.0091	0.0009	10.74***	0.0245	0.0007	33.95***
Dispersion in hourly wages (lngap2)	−0.0654	0.0317	−2.06**	0.1011	0.0154	6.57***
Dispersion in total income (lngap3)	0.1289	0.0313	4.12***	0.1762	0.0159	11.08***

*** p < 0.01, ** p < 0.05, * p < 0.1.

Source: Own elaboration base on ECH 1988 and 1996.

**Table 14:** **Placebo using as control group all the public workers (emp_pub1), 1988-1996**

Outcome variable	Coefficient	Standard error	z
Monthly earnings	0.0663	0.0137	4.84***
Hourly labor earnings (monetary)	0.0493	0.0136	3.63***
Hourly wages	0.0512	0.0133	3.84***
Hourly labor earnings	0.0512	0.0132	3.87***
Unemployment probability	0.0206	0.0005	39.07***
Dispersion in hourly wages (lngap2)	0.0710	0.0137	5.2***
Dispersion in total income (lngap3)	0.1771	0.0138	12.82***

*** p < 0.01, ** p < 0.05, * p < 0.1.

Source: Own elaboration base on ECH 1988 and 1996.

These results would indicate that more educated people and men in particular, are the ones to win the most in face of increased trade liberalization.

Finally, we perform one last exercise: we analyze the effects for several years from 1988–1992 onwards. We report the results in Table 15. We find that for 1988–1992 (one year after Mercosur’s creation) a higher impact in earnings variables, a lower probability of unemployment and not significant hourly wage dispersion and similar total wage dispersion compared to the period 1988–1996. Nevertheless, for the period 1988–1993 we find some unexpected results: mainly a reduction in all the outcome variables considered. The reduction in earnings variables could be explained due to the expiration of the contracts signed under the Wage Council system, and the fall in union density in 1993. Eventually, it could be argued that the fall in unemployment probability could be a result of a more flexible labor market. It is harder to pose an explanation for the fall in wage dispersion. One possible explanation could be by the reduction in the inflation levels in 1993 as a consequence of the stabilization plan implemented in 1992.

**Table 15:** **For several years double-robust estimates for time periods**

Time period							
Outcome variable	88-92	88-93	88-94	88-95	88-96	88-97	88-98
Monthly earnings	0.1749	−0.0211	0.1249	0.1056	0.0663	0.0075	0.0620
	(0.0112)***	(0.0112)	(0.0124)***	(0.0146)***	(0.0137)***	(0.0137)	(0.0151)***
Hourly labor earnings (monetary)	0.1565	−0.0408	0.1098	0.1021	0.0493	0.0001	0.0611
	(0.0110)***	(0.0111)***	(0.0122)***	(0.0174)***	(0.01356)***	(0.0132)	(0.0142)***
Hourly wages	0.1385	−0.0410	0.0983	0.0952	0.0512	−0.0262	0.0335
	(0.0112)***	(0.0107)***	(0.0122)***	(0.0182)***	(0.0133)***	(0.0142)	(0.0139)**
Hourly labor earnings	0.1646	−0.0253	0.1375	0.1333	0.0512	0.0089	0.0841
	(0.0119)***	(0.0111)**	(0.0121)***	(0.0173)***	(0.0132)***	(0.0127)	(0.0138)***
Unemployment probability	0.0041	−0.0010	0.0040	0.0123	0.0206	0.0308	0.0178
	(0.0005)***	(0.0005)**	(0.0005)***	(0.0005)***	(0.0005)***	(0.0005)***	(0.0005)***
Dispersion in hourly wages	0.0008	−0.1460	0.0340	−0.0503	0.0103	−0.0639	−0.0293
(lngap2)	(0.0110)	(0.0111)***	(0.0122)***	(0.0176)***	(0.0137)	(0.0128)***	(0.0142)**
Dispersion in total income	0.1662	−0.0590	0.1685	0.1498	0.1771	0.0532	0.1663
(lngap3)	(0.0113)***	(0.0113)***	(0.0125)***	(0.0147)***	(0.0138)***	(0.0132)***	(0.0151)***

*** p < 0.01, ** p < 0.05, * p < 0.1. Standard errors in parenthesis.

Source: Own elaboration base on ECH 1988 and 1996.

For the periods 1988–1994 and 1988–1985 we observe again an important increase in wages, unemployment and total wage dispersion, while hourly wage dispersion shows a positive increase in the period 88–94 but then reverses its sign in 1988–1995.

Finally, for the periods 1988–1996 –commented above-, 1988–1997 and 1988–1998 there is also a positive effect on earnings but lower than for the previous years –with the exception of 1988-1993-, increases in unemployment probability, and qualitatively similar results for total earnings dispersion, while hourly wage dispersion changed sign.

Thus, except for 1988–1993 and for the outcome variable hourly wage dispersion, results are qualitatively similar to those found five years after Mercosur’s creation.

## Consistency checks

Since there were important changes in the methodology used by the INE in 1991, such as changes in the sampling and the questionnaires applied we try also 1991 as the baseline year and 1996 as the year after the intervention. We report the results in Tables 16 and 17.

**Table 16:** **Robustness tests: results of the estimation of regression equations in double differences**

Variables	Monthly labor earnings	Hourly labor earnings (monetary)	Hourly wages	Hourly labor earnings (monetary and in-kind)	Unemployment probability
Treatment	1.489***	0.0123	0.0498***	0.0417***	0.0245***
	(0.0161)	(0.0131)	(0.0131)	(0.0127)	(0.000569)
Montevideo	0.118***	0.230***	0.207***	0.205***	0.00693***
	(0.0178)	(0.00996)	(0.0100)	(0.00971)	(0.000450)
Sex	0.235***	0.180***	0.179***	0.186***	−0.0289***
	(0.0228)	(0.0134)	(0.0130)	(0.0126)	(0.000701)
Age	0.0775***	0.0467***	0.0434***	0.0443***	−0.0179***
	(0.0055)	(0.00314)	(0.00309)	(0.00302)	(0.000185)
Age2	−0.00070***	−0.000432***	−0.000356***	−0.000398***	0.000186***
	(6.95e-05)	(3.93e-05)	(3.87e-05)	(3.79e-05)	(2.14e-06)
Head	0.174***	0.143***	0.0846***	0.107***	−0.0397***
	(0.0235)	(0.0132)	(0.0132)	(0.0126)	(0.000595)
Education	0.0919***	0.0655***	0.0762***	0.0677***	−0.00442***
	(0.00261)	(0.00144)	(0.00141)	(0.00138)	(7.11e-05)
Marital status	0.0919***	0.103***	0.0728***	0.0883***	−0.0188***
	(0.0212)	(0.0117)	(0.0115)	(0.0112)	(0.000481)
Hours worked	0.007***				−2.02e-05
	(0.00073)				(1.87e-05)
Number of works	−0.059**	0.0152***	0.0165**	0.0162**	0.000363
	(0.0054)	(0.00570)	(0.00771)	(0.00769)	(0.000348)
Constant	3.464***	1.835***	1.598***	1.799***	0.550***
	(0.110)	(0.0602)	(0.0589)	(0.0580)	(0.00412)
Observations	12,182	12,182	12,133	12,164	12,182
R-squared	0.360	0.307	0.326	0.305	0.882

Robust standard errors in parenthesis; *** p < 0.01, ** p < 0.05, * p < 0.10.

Source: Own elaboration base on ECH 1991 and 1996.

**Table 17:** **Results of the estimation of matching and difference-in-differences with double-robust estimates and inverse probability weighting, 1991-1996**

	Coefficient	Standard error	z
Monthly labor earnings	1.428	0.017	85.41***
Total hourly labor earnings	0.045	0.014	3.23***
Hourly wages (monetary)	0.087	0.013	6.48***
Hourly labor (monetary and in-kind)	0.081	0.013	6.23***
Unemployment Probability	0.022	0.001	40.42***
Dispersion in hourly wages (GAP2)	0.065	0.065	4.53***
Dispersion in total earnings (GAP3)	0.209	0.015	14.28***

*** p < 0.01, ** p < 0.05, * p < 0.10.

Source: Own elaboration base on ECH 1991 and 1996.

For the DID regressions we find a positive significant impact of Mercosur’s creation for monthly labor earnings, hourly labor earnings, hourly wages (monetary), and hourly labor earnings in money and in kind, though the estimated coefficient differs from previous results. Further, increased trade openness seems to increase the probability of unemployment. The only difference is that monetary hourly wages turns out to be positively significant taking 1991 as baseline year, while it is was not significant when we take 1988 as baseline.

Also income and wage dispersion shows a similar behavior taking 1991 as baseline instead of 1988.

As we commented above Barraud (2009) implemented a difference-in-difference approach using household surveys for the years 1988 and 1998, for the Argentinean case. Barraud define as treated workers in manufacturing industries and as control the public employees. The main findings were a reduction in monthly and hourly earnings and increases in the probability of unemployment. Nevertheless for the Uruguayan case we find increases in wages and also in the probability of unemployment and a rise in wage dispersion.

## Concluding remarks

The results for the difference-in-difference regressions seem to show that increased trade openness due to Mercosur’s creation has had a positive impact on total earnings of the treated group, i.e. manufacturing workers, but when controlling for hours worked it is not significant which could be pointing out a countervailing effect due to the hours worked, i.e. the higher income is due to more hours worked and not a higher hourly wage. Furthermore, we observe that increased trade openness impact positively and significantly on the probability of unemployment of the treated group. Finally, we observe a positive effect on monthly earnings (in monetary terms plus in kind) and increases for our three measures of wage dispersion tried.

Since these results could be affected by differences in the characteristics of individuals in the treated and control group we apply double robust estimators finding significant increases in income and hourly wages as well in the probability of unemployment and increased wage dispersion. Thus, our results confirms the findings by Casacuberta and Vaillant (2002) and Galiani and Sanguinetti (2003) who working at the industry level find that Mercosur trade flows have negatively affected the level of industry employment in Uruguay, but opposite to the findings by Casacuberta et al. (2002) regarding to the impact on wages. However their results are obtain through correlations so they do not have a causal interpretation. In this regard we contribute to the literature providing a causal nexus between Mercosur’s creation and labor market outcome and could give us an insight of the effects of new free trade agreements with relatively more developed countries or blocs. In our research agenda is to dig deeper into the relations between trade and labor market outcomes, and if possible to work with matched employees and firm level data, including as well into the analysis institutional factors and technological change.

## Endnotes

^a^ For a survey on the theoretical links of globalization and inequality see Goldberg and Pavcnik (2007).

^b^ A large number of multi-country case studies and econometric studies using cross-country datasets have tested the empirical validity of the trade-growth relationship but there is no full agreement among economists concerning the precise nature of this relationship(Baldwin 2004; Rodriguez y Rodrik 2000; Dollar y Kraay 2004;; Loayza, Fajnzylber, y Calderón Loayza, Fajnzylber, y Calderón ; Loayza, Fajnzylber, y Calderón 2004; Wacziarg y Welch 2003).

^c^ For a recent theoretical model with unemployment see Helpman, Itskhoki and Redding (2010).

^d^ Winters et al. (2004) and Hertel and Reimer (2005) surveyed the effects of trade on income levels.

^e^ The tariff schedule in place before trade liberalization may also affect the impact of trade on wage inequality. If protection was higher in the low-skill intensive sectors, then trade liberalization may actually lead to shrinkage of these sectors.

^f^ Trefler (2001) for the Canada-US Free Trade Agreement, uses difference-in-differences estimators finding an important role of tariffs cuts in employment reduction, but important productivity improvement and that it takes a seven year period for wages to increase after trade liberalization.

^g^ Uruguay belongs to the called “early reformers”, that is to say the countries who first apply the first-generation reforms in Latin America, including an important financial liberalization.

^h^ The Asuncion Treaty, signed on the 26th March of 1991 is a regional integration agreement to create the Southern Common Market. It was signed by Argentina, Brazil, Paraguay and Uruguay.

^i^ Source: Banco Central del Uruguay

^j^ The exchange rate policy has consequences on the domestic currency appreciation and through this channel to trade specialization.

^k^ Furthermore, the percentage of unemployment in manufacturing for 1996 is of 12.52% while in the control group is 7.5%.

^l^ A technique to address contagion is to apply general equilibrium models, but they also are not free of calibration issues.

^m^ Blundell and Costa Dias (2000) present a review of the microeconomic evaluation literature.

^n^ According to the definition of the outcome variables in this work, both groups exclude those who declare to have worked zero hours in the week previous to the survey and to those who do not declare income in the month previous to the survey.

°See the Appendix for details on the estimation of the probability of unemployment.

^p^ Since earnings are expressed in a monthly basis we divide the total monthly earnings by 4.3 to obtain the weekly income. Monthly earnings and hourly labour earnings include leave pays and bonuses.

^q^ Using the average value of the 80th percentile protects us against outliers.

^r^ Lack of data prevents us from analyzing wage dispersion at the 3-digit ISIC level.

^s^ Available upon request.

^t^ See Appendix for details.

^u^ Confounding occurs when there are variables that can affect the outcome of interest, which are correlated to the treatment under analysis. Confounding as well as not common trends can be controlled for using inverse probability weighting.

^v^ The balancing tests are available upon request.

^w^ See Table 5 for the definition of the various control groups.

## Appendix

### Probability of unemployment

In this section we provide details on the estimation of unemployment probability and its behavior for control and treated groups.

To estimate the probability of unemployment we first use a logit model, where Pr(y = 1|X) = F(X), where ***y*** is the probability of unemployment and ***X*** are the covariates: Montevideo, Sex, age, squared age, head of household, education and marital status. Results are reported in Table 18. After estimating the $\hat{\beta }$ we predict the probability of unemployment for each individual. In Table 19 we present the marginal effects evaluated at the mean.

**Table 18:** **Probability of unemployment**

	1988	1996
Montevideo	0.153***	0.219***
	(0.0467)	(0.0412)
Sex	−0.676***	−0.391***
	(0.0529)	(0.0448)
Age	−0.202***	−0.141***
	(0.0129)	(0.0110)
Age^2	0.00202***	0.00134***
	(0.000166)	(0.000141)
Head of household	−0.422***	−0.697***
	(0.0706)	(0.0591)
Schooling years	−0.0364***	−0.0945***
	(0.00672)	(0.00647)
Marital Status	−0.302***	−0.176***
	(0.0550)	(0.0475)
Constant	2.629***	2.224***
	(0.222)	(0.192)
Observations	28,978	27,752
Pseudo R2	0.112	0.0945

*** p < 0.01, ** p < 0.05, * p < 0.10.

Source: Own elaboration base on ECH.

**Table 19:** **Marginal effects evaluated at the mean**

**1988**	**dy/dx**	**Std. Err.**	**z**	**P > z**	**[95% Conf.**	**Interval]**	**Mean**
Montevideo	0.00791	0.00242	3.27	0.001	0.0032	0.0127	0.4585
Sex	−0.03506	0.00273	−12.85	0	−0.0404	−0.0297	0.5896
Age	−0.01047	0.00069	−15.27	0	−0.0118	−0.0091	38.0812
Age^2	0.00010	0.00001	11.92	0	0.0001	0.0001	1610.8420
Head	−0.02188	0.00359	−6.1	0	−0.0289	−0.0148	0.4755
Schooling years	−0.00189	0.00035	−5.44	0	−0.0026	−0.0012	8.5182
Marital status	−0.01565	0.00284	−5.5	0	−0.0212	−0.0101	0.6524
**1996**	**dy/dx**	**Std. Err.**	**z**	**P > z**	**[95% Conf.**	**Interval]**	**Mean**
Montevideo	0.01685	0.00317	5.32	0	0.0106	0.0231	0.5259
Sex	−0.03012	0.00343	−8.79	0	−0.0368	−0.0234	0.5590
Age	−0.01089	0.00085	−12.76	0	−0.0126	−0.0092	38.0718
Age^2	0.00010	0.00001	9.43	0	0.0001	0.0001	1612.9000
Head	−0.05366	0.00441	−12.18	0	−0.0623	−0.0450	0.4367
Schooling years	−0.00727	0.00048	−15	0	−0.0082	−0.0063	9.2244
Marital status	−0.01353	0.00365	−3.71	0	−0.0207	−0.0064	0.6084

Source: Own elaboration base on ECH.

In the period the variables that change most the marginal effect were for individuals living in Montevideo with an elasticity of 0.0079 in 1988 and of 0.016 in 1996, and for years of schooling that was of −0.002 in 1988 and of −0.007 in 1996.

For both years, 1988 and 1996, we analyze if the probability of unemployment were similar between the treatment and the control group by means of a *t*-test finding that in fact the probability of unemployment is higher in the manufacturing sector than in the control group. We present the results for 1988 in Table 20, in which the difference between both groups is of 0.03. The same feature is observed for 1996 but with a slight increase in unemployment for the treated group (Table 21), and the difference between both groups is of 0.04.

**Table 20:** **Probability of unemployment, two-sample** ***t*** **test with equal variances by group (treatment group = 1 and control group = 0; year1988)**

Group	Obs	Mean	Std. Err.	Std. Dev.	[95% Conf. Interval]	Group
0	3526	0.0551943	0.000808	0.047978	0.0536101	0.0567784
1	3648	0.0874021	0.0013138	0.079354	0.0919938	0.089978
combined	7174	0.0715721	0.0008001	0.0677654	0.0700037	0.0731404
diff		−0.0322079	0.0015546		−0.0352554	−0.0291603

diff = mean(0) - mean(1); t = −20.7173; Ho: diff = 0; degrees of freedom = 7172.

**Table 21:** **Probability of unemployment, Two-sample** ***t*** **test with equal variances for treatment = 1 and control group = 0, 1996**

Group	Obs	Mean	Std. Err.	Std. Dev.	[95% Conf. Interval]	Group
0	3041	0.0748768	0.001002	0.0552579	0.072912	0.0768415
1	2625	0.115858	0.001657	0.0848971	0.1126088	0.1191072
combined	5666	0.093863	0.0009758	0.0734488	0.0919501	0.0957758
diff		−0.0409813	0.0018797		−0.0446662	−0.0372963

diff = mean(0) - mean(1); t = −21.8020; Ho: diff = 0; degrees of freedom = 5664.

In 1996 for both groups there is a higher probability of unemployment than in 1988, before Mercosur´s creation as can be seen in the Table 22.

**Table 22:** **Probability of unemployment, two-sample** ***t*** **test with equal variances by treatment (intervencion)**

Group	Obs	Mean	Std. Err.	Std. Dev.	[95% Conf. Interval]	Group
0	7530	0.0692962	0.0007144	0.0619908	0.0678958	0.0706966
1	5666	0.0938942	0.0009694	0.072972	0.0919938	0.0957947
combined	13196	0.0798579	0.0005922	0.0680229	0.0786972	0.0810186
diff		−0.024598	0.001177		−0.0269052	−0.0222909

diff = mean(0) - mean(1); t = −20.8985; Ho: diff = 0; degrees of freedom = 13194.
